# Molecular Mechanisms Responsible for Diastolic Dysfunction in Diabetes Mellitus Patients

**DOI:** 10.3390/ijms20051197

**Published:** 2019-03-09

**Authors:** Jovana Nikolajević Starčević, Miodrag Janić, Mišo Šabovič

**Affiliations:** Department of Vascular Diseases, University Medical Centre Ljubljana, Zaloška cesta 7; SI-1000 Ljubljana, Slovenia; jovana_nikolajevic@yahoo.com (J.N.S.); miodrag.janic@kclj.si (M.J.)

**Keywords:** heart failure, diabetes, diabetic cardiomyopathy, diastolic dysfunction, mechanisms

## Abstract

In diabetic patients, cardiomyopathy is an important cause of heart failure, but its pathophysiology has not been completely understood thus far. Myocardial hypertrophy and diastolic dysfunction have been considered the hallmarks of diabetic cardiomyopathy (DCM), while systolic function is affected in the latter stages of the disease. In this article we propose the potential pathophysiological mechanisms responsible for myocardial hypertrophy and increased myocardial stiffness leading to diastolic dysfunction in this specific entity. According to our model, increased myocardial stiffness results from both cellular and extracellular matrix stiffness as well as cell–matrix interactions. Increased intrinsic cardiomyocyte stiffness is probably the most important contributor to myocardial stiffness. It results from the impairment in cardiomyocyte cytoskeleton. Several other mechanisms, specifically affected by diabetes, seem to also be significantly involved in myocardial stiffening, i.e., impairment in the myocardial nitric oxide (NO) pathway, coronary microvascular dysfunction, increased inflammation and oxidative stress, and myocardial sodium glucose cotransporter-2 (SGLT-2)-mediated effects. Better understanding of the complex pathophysiology of DCM suggests the possible value of drugs targeting the listed mechanisms. Antidiabetic drugs, NO-stimulating agents, anti-inflammatory agents, and SGLT-2 inhibitors are emerging as potential treatment options for DCM.

## 1. Introduction

The incidence of diabetes mellitus (DM) is continuously growing, threatening to become one of the most important health problems in the near future worldwide. Whereas the micro- and macrovascular complications have been extensively studied in the last decades, the importance of heart failure (HF) in diabetic patients has only recently attracted researchers’ attention. Epidemiologic studies are showing that prevalence of HF in diabetic patients is very high, being, at least, ~30% [1,2]. HF is twice as prevalent in diabetic men and up to 6 times more prevalent in diabetic women, than in a comparable nondiabetic population [3,4]. Studies also consistently show that HF is a major cause of hospitalizations and important predictor of increased mortality in diabetic patients [5,6].

Diabetic patients are commonly affected by a specific type of cardiomyopathy, named diabetic cardiomyopathy (DCM), in order to emphasize its specific etiology and presentation, and to distinguish it from other forms of cardiomyopathy. According to the European Society of Cardiology and European Association for the Study of Diabetes guidelines, DCM is defined as ventricular dysfunction that occurs in the absence of coronary atherosclerosis and hypertension in diabetic patients [7]. The prevalence of DCM is estimated to be ~1.1% in general population and 16.9% in diabetic population [8]. It should be kept in mind that diabetic patients also suffer from other types of cardiomyopathy, such as ischemic or hypertensive cardiomyopathy that have different underlying pathophysiology and clinical course. 

Clinically, DCM presents as heart failure with preserved ejection fraction (HFpEF). Myocardial hypertrophy and diastolic dysfunction have been considered the hallmarks of DCM, whereas systolic function is affected in the latter stages of the disease [9]. Structural myocardial changes can be detected early in the disease course, even before its first clinical manifestation. The first visible structural change is slight myocardial hypertrophy accompanied by perivascular and interstitial fibrosis as well as collagen deposition [10,11]. In addition to macroscopic remodeling, cardiomyocytes also undergo microscopic and ultrastructural changes [12]. 

Different imaging tools could be used for diagnosis of DCM based on morphological characteristics and assessment of cardiac function. Echocardiography is the most widely used imaging modality for assessment of cardiac morphology as well as systolic and diastolic function due to its availability and low cost. Diastolic function is determined by using pulse wave doppler for assessing transmitral and pulmonary venous flow, tissue doppler imaging (TDI) for assessing myocardial tissue velocities during the cardiac cycle, and measuring of the left atrial volume [13]. It enables not only an estimation of diastolic function but also a follow-up of disease progression from mild diastolic dysfunction (impaired relaxation) to advanced stages (pseudonormalization or restriction). Cardiac magnetic resonance imaging (CMRI) has recently emerged as a useful imaging tool for diagnosing various structural and functional disorders of the myocardium including diastolic dysfunction and myocardial steatosis. Due to their capability to detect myocardial metabolic abnormalities, CMRI and positron emission tomography (PET) may be useful in the diagnosis of DCM [14]. 

In this review we have tried to elucidate the mechanism of DCM in a somewhat different way, i.e., by constructing a new model that describes the underlying mechanisms and their connections, all responsible for the development of diastolic dysfunction. This is made possible through knowledge gained from important recent data from new clinical and basic studies as well as through translation of previous knowledge and concepts of vascular pathology to the myocardial pathology in diabetic patients. We believe that similar fundamental processes underlying myocardial and vascular pathology in these patients exist.

## 2. Pathophysiological Process Underlying Diabetic Cardiomyopathy

**Figure 1:**
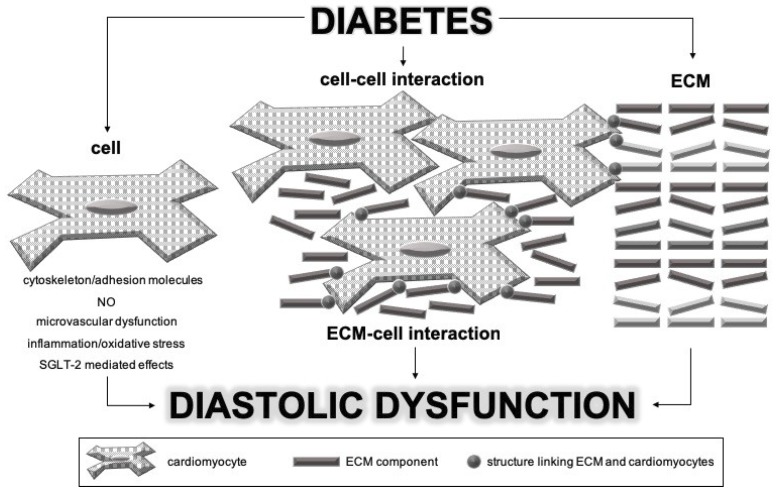
Pathophysiological mechanisms involved in the pathogenesis of diabetic cardiomyopathy. SGLT-2—sodium glucose cotransporter-2; NO—nitric oxide; ECM—extracellular matrix.

Different parts of myocardium are involved in the pathophysiological process of DCM: cardiomyocytes, extracellular matrix (ECM), and interactions between and among them. Based on the knowledge of vascular wall involvement in vascular stiffness in diabetic patients, we propose that cardiomyocytes probably have a pivotal role in inducing the process of myocardial stiffening. Namely, we believe that investigations of complex mechanisms should start with identification of the initial pathological process. The proposed mechanism of DCM development and progression is illustrated in Figure 1. 

As mentioned above, hypertrophy is one of the first visible structural changes in DCM. DM itself promotes cardiomyocyte hypertrophy and contractile dysfunction as well as reduction in their number [15]. Cardiomyocyte number reduction and consequent hypertrophy could be the result of activated apoptotic mechanisms and reduced proliferation potential, hypertrophy being compensatory in this setting [16]. 

The first functional change is cardiomyocyte stiffening due to their increased tone and impairment of their cytoskeleton, both of which are induced by DM itself. Several other mechanisms, specifically affected by DM, seem to be significantly involved in increasing cardiomyocytes’ stiffness: (i) impairment of myocardial nitric oxide (NO) pathway, (ii) coronary microvascular dysfunction, (iii) increased inflammation and oxidative stress, and (iv) myocardium-sodium glucose cotransporter-2 (SGLT-2) related defects. 

Cardiomyocytes with increased intrinsic cell stiffness change their phenotype to more fibrogenic. Hyperglycemia may also induce this fibrogenic phenotype independently of the intrinsic mechanisms [17]. Fibrogenic phenotype of cardiomyocytes is characterized by increased synthesis and release of cytokines that induce fibroblast proliferation and activation as well as proinflammatory mediators that trigger fibrosis through activation of immune cells: angiotensin II, transforming growth factor β (TGFβ), tumor necrosis factor α (TNFα), and interleukin-1 [17].

ECM has an important role in preservation of myocardial architecture and chamber geometry [18]. Collagen fibers weave during systole and uncoil during diastole thus maintaining cardiomyocyte alignment. Once the fibers straighten, they resist further expansion and protect cardiomyocytes from overstretching [19]. As previously stated, myocardial ECM in patients with DCM expresses microscopic changes such as perivascular and interstitial fibrosis as well as collagen deposition, especially collagen types I and III [10,11,20]. This is predominantly the result of fibrogenic cardiomyocyte phenotype, which in turn stimulates the fibroblasts to produce more collagen. The relationship between myocardial stiffness and collagen content is not linear, suggesting contribution of additional factors in stiffness development [21]. In addition to total amount of collagen and expression of collagen type I, myocardial stiffness also depends on the degree of collagen cross-linking [18,22]. In diabetic patients, high levels of advanced glycation end-products (AGEs) contribute to fibrosis and diastolic stiffening by increasing the level of collagen cross-linking [23]. Cross-linking of collagen molecules prevents their enzymatic degradation leading to increase in collagen amount in ECM [24]. 

Due to different pathophysiological mechanisms, we think that it is possible to differentiate between cell stiffness and ECM stiffness. Changes in matrix stiffness change cell–matrix interactions and activate cellular mechanoreceptors, thus further increasing intrinsic cell stiffness and promoting its secretory phenotype. This pathological positive feedback loop (cell stiffness–matrix stiffness–cell stiffness) is closed, leading to diastolic dysfunction and eventually heart failure. All pathophysiological mechanisms mentioned above are further discussed in detail.

## 3. Models of Diabetic Cardiomyopathy

In order to evaluate and explore the mechanisms of DCM, several animal models were introduced. Animals used in the studies develop diabetes that is either diet/drug-induced or transgenic [25]. Animal models provide good base for the study of DCM, but there are some differences based on animal types and the level of insulin resistance/depletion [17]. 

Type 1 DM can be induced by streptozotocin (STZ), a drug that, in high doses, destructs pancreatic β-cells, thus causing insulin-dependent state. It is used in rodent models. Animals develop accelerated myocardial fibrosis, cardiomyocyte hypertrophy, accelerated cell death, decrease in cardiomyocyte contractile function, and left ventricular hypertrophy [25,26,27]. Similar effects, with myocardial fibrosis and collagen accumulation, have also been demonstrated in large animal models, such as mongrel dogs and rhesus monkeys, where insulin-dependent diabetes was induced by alloxan administration [17,28,29]. On the other hand, transgenic mice, the so-called Akita mice, developed diastolic dysfunction and lipotoxicity without significant cardiac fibrosis and hypertrophy [30]. Other transgenic mice models of type 1 DM have also been used, namely OVE26 and non-obese diabetes (NOD) mice [25].

Type 2 DM animal models consist particularly of rodents, where insulin resistance/depletion is induced either by diet or are transgenic [17]. Diet-induced animal models comprise of mice fed with either high fat diet (HFD) or the so-called “Western” diet. The myocardial changes in these animals are subtle and take longer to develop [25]. Nevertheless, after certain amount of time, ventricular hypertrophy, myocardial fibrosis, and decreased cardiac contractile function develop [25,31]. Commonly used type 2 DM transgenic models consist of obesity (ob/ob) and insulin resistance, as well as type 2 DM (db/db) models. These are usually mice with truncated leptin receptor or fully leptin deficient. Therefore, they are resistant to central leptin effects. These mice become fat in relatively short time, and show signs of insulin resistance and/or diabetes in further course [17]. Cardiac fibrosis, accompanied by left ventricular hypertrophy and diastolic dysfunction, has been observed in these models [32]. It is important to note that the number of pathological features in these type 2 DM animal models is mostly dependent on diabetes duration, i.e., observation/follow-up period. Zucker diabetic fatty (ZDF) and Goto-Kakizaki (GK) rats largely develop similar features, as described previously [17,25]. Direct animal models of DCM are only transgenic and include mice with cardiomyocyte specific overexpression of the transcription factor for peroxisome proliferator activated receptor α (MHC-PPARα) and cardiomyocyte selective insulin receptor knockout (CIRKO) mice. Their hearts show similar characteristics as the hearts of type 2 DM patients, particularly expressing diastolic dysfunction and reduced cardiac contractility [25,33,34,35].

As pathological features of DCM are similar in humans and animal models, the latter allow for research of mechanistic pathways as well as therapeutic targets.

## 4. Diabetes Mellitus and Increased Intrinsic Cardiomyocyte Cell Stiffness

Morphology of cardiomyocytes is changing on both macro- and microscopic levels during the course of DM. Histological examination of cardiomyocytes from diabetic mice showed irregular nucleus size as well as fragmentation of actin fibers and diffuse and irregular actin deposition especially in cortical regions [36]. Even more important is the fact that cardiomyocytes in DM change their mechanical properties. Several parameters used to analyze changes of mechanical characteristics of diabetic cardiomyocytes confirmed that increased cellular stiffness is an important factor in myocardial stiffness and that resting tension (RT) is defined as the passive force at the same sarcomere length. Increased RT is in correlation with increased myocardial stiffness [37,38] and has been reported in animal models to be associated with both aging and obesity [39,40]. Cardiomyocytes isolated from diabetic mice expressed increased RT compared to those from nondiabetic mice [37,38]. 

According to available data, cytoskeleton changes contribute substantially to increased cellular stiffness observed in DM. The cytoskeleton consists of complex network of filaments and tubules that transmit mechanical and chemical stimuli within and between cells. The cytoskeleton is also involved in maintaining cell stability by organizing the cell content [41]. Atomic force microscopy (AFM) is increasingly being used to measure cytoskeletal components as well as viscoelastic properties of living cells. This method enables quantification of changes in the myocyte sarcolemma, sarcomeric skeleton and cytoskeletal proteins including actin, tubulin, and titin. On the other hand, it allows measurement of elastic modulus of living cells [42,43]. A recent study using AFM showed that the elastic modulus of diabetic cardiomyocytes was significantly increased compared to nondiabetic ones. They also observed changes in actin organization, diffuse irregular actin deposition, and disordered and broken actin filaments in diabetic cardiomyocytes. They concluded that changes in intrinsic mechanical properties were probably not related to the contractile state of the cell proteins, but rather to direct changes of material properties caused by diabetes itself. The same study also revealed higher adhesive force in diabetic cardiomyocytes than in nondiabetic ones, suggesting increased number and/or activation state of adhesion molecules on the cell surface [36].

Normal functioning of cardiomyocytes depends on the precise control of Ca^2+^ levels during the contraction–relaxation cycle [44]. Depolarization of cardiomyocyte membrane leads to opening of voltage-dependent L-type Ca^2+^ channels allowing Ca^2+^ ions to enter the cell. The increasing intracellular level of Ca^2+^ triggers further Ca^2+^ release from sarcoplasmic reticulum (SR) via Ca^2+^ release channels (ryanodine receptors). Lowering of cytoplasmic concentration of Ca^2+^ allows cardiomyocyte relaxation in diastole. During relaxation Ca^2+^ concentration returns to diastolic level mainly by reuptake of Ca^2+^ to SR by sarco/endoplasmic reticulum Ca^2+^-ATPase 2 (SERCA2) as well as the sarcolemmal Na^+^-Ca^2+^ exchanger and the sarcolemmal Ca^2+^ ATPase. SERCA2 has the most important role in maintaining Ca^2+^ levels during relaxation as it accounts for approximately 70% of the reuptake of Ca^2+^ [45].

Expression of the SERCA2 is decreased in DM due to glucose toxicity and increased oxidative stress [36,46,47]. Reduced SERCA2 expression leads to decrease of recaptured Ca^2+^ and lowering of SR Ca^2+^ content. Consequently, less Ca^2+^ is available for the next contraction causing impaired cardiomyocyte contractility. On the other hand, increased cytoplasmic Ca^2+^ level during diastole leads to impaired cardiomyocyte relaxation observed in DCM [46,48]. However, some authors proposed that increased cell stiffness is independent from membrane Ca^2+^ channels and cytosolic Ca^2+^ concentration, since increased cardiomyocyte stiffness was confirmed at different intracellular Ca^2+^ levels [36]. This suggests that disordered cytoskeletal organization caused by DM itself is a fundamental process underlying increased cell stiffness in DCM. 

Structural and functional changes in titin—a sarcomeric protein that acts as a molecular spring, maintains sarcomere stability, and determines passive myofilament distensibility—are also directly affected by DM. Mechanical properties of titin depend on its isoform composition as well as post-translational modifications such as phosphorylation and disulfide bonding caused by oxidative stress [49]. Two titin isoforms are present in the cardiac muscle: the smaller N2B isoform, which is constituted only of N2B element, and the larger N2BA isoform, which contains both N2B and N2A elements. In human cardiomyocytes the N2BA:N2B isoform ratio is approximately 30:70. Cardiomyocytes from diabetic patients express higher N2BA:N2B titin isoform ratio, which is considered more compliant than those found in nondiabetic cardiomyocytes. Nevertheless, titin-based passive tension of diabetic cardiomyocytes is increased compared to nondiabetic ones. This is probably due to altered titin phosphorylation caused by both insulin deficiency/impaired insulin signaling and increased disulfide bonding caused by increased oxidative stress in DM [50]. 

## 5. Nitric Oxide and Increased Contractile State of Cardiomyocytes in Diabetes

NO signaling is thought to be impaired and directly implicated in the pathophysiology of DCM. NO is formed by NO synthase (NOS), which has three isoforms that are all present in cardiomyocytes: endothelial NOS (eNOS), neuronal (nNOS), and inducible (iNOS). These are compartmentalized in different parts of cardiomyocytes [51,52]. eNOS is localized in the caveolae and responsible for constitutive production of NO that reduces heart rate, contraction and oxygen consumption while increasing diastolic relaxation. Additionally, it has an antiapoptotic action and inhibits aggregation and adhesion of platelets. Its vasodilatory action is mediated through guanylate cyclase activation. Neuronal NOS is located at the sarcoplasmic reticulum where it regulates several receptor activities, namely SERCA2, ryanodine receptor, and the L-type Ca^2+^ channel. Under basal conditions, eNOS and nNOS are responsible for NO production that attenuates inotropic responsiveness and promotes relaxation of the cardiomyocyte. These NO functions are protective. eNOS activity is typically reduced in patients with diabetes, leading to the lack of NO [53]. In rare cases, NO can also play a detrimental role when it is present in excess amounts in the cardiomyocytes. In situations like hyperglycemia, oxidative stress, and hyperinsulinemia, iNOS can additionally produce large toxic amounts of NO. High amounts of NO lead to contractile dysfunction, while in the case of substrate limitation, iNOS can be uncoupled and starts to produce reactive oxygen and nitrate species. This leads to the environment of high oxidative stress and inflammation, causing direct tissue damage on one hand and, on the other hand, the nitration of actin and other cytoskeleton proteins and channels, altering their structure and having detrimental effect on the contractile function. Similar eNOS uncoupling has also been described in diabetic myocardium, also with destructive effects [51,53,54]. Summing up, NO has a very important role in the diabetic myocardium. While in controlled limited amounts, it acts protectively, allowing for efficient cardiomyocyte relaxation, on the other hand, when it is exhausted or in excess, it makes the microenvironmental shift that causes switch to “reduced relaxation-increased contraction” state of cardiomyocytes, thus contributing to increased myocardial stiffness. 

## 6. Inflammation and Oxidative Stress in Cardiomyocytes in Diabetes

DM is considered to be a complex metabolic disorder, characterized by increased levels of oxidative stress and inflammation. It has been widely accepted that complex relationship between oxidative and nitrosative stress and proinflammatory mechanisms plays a very important role in the development of micro- and macrovascular diabetic complications. Better understanding of DCM pathophysiology led to the realization that these mechanisms also participate in the pathogenesis of DCM.

Hyperglycemia is considered to be the most important factor causing increased oxidative stress in DM. Hyperglycemia, accompanied by insulin resistance and hypertriglyceridemia, limits cardiomyocytes’ ability to use glucose as an energy source, increasing the utilization of free fatty acids (FFAs) and leading to increased production of reactive oxygen species (ROS) [55]. The major sources of ROS production in myocardium are mitochondria in cardiomyocytes, endothelial cells and neutrophils [56]. Non-mitochondrial sources, including nicotinamide adenine dinucleotide phosphate (NADPH) oxidase, xanthine oxidase, and microsomal P-450 enzyme activity, could also substantially contribute to increased ROS production [57]. Increased levels of ROS could lead to cellular damage through several mechanisms, including oxidative modification of proteins, modulating the production and function of NO, as well as modulation of intracellular signaling pathways leading to cellular hypertrophy, apoptosis, and necrosis [58,59]. Increased oxidative stress in the myocardium is associated with oxidative modification of proteins involved in contractility, excitation–contraction coupling, protein folding, antioxidant defense, fatty acid and glucose metabolism, and Ca^2+^ handling [60]. Finally, oxidative damage could also alter the architecture of the ECM by activating matrix metalloproteinases and promoting formation of AGEs [57,61]. 

Apoptosis and necrosis of cardiomyocytes and endothelial cells are also important features of DCM, leading to reduced number of cardiomyocytes and expansion of extracellular space [62]. Apoptosis does not cause scar formation and accumulation of interstitial collagen, while necrosis is associated with widening of extracellular space and increased deposition of collagen leading to increased fibrosis and ECM stiffness [63].

Hyperglycemia is also responsible for chronic low-grade inflammation, which is commonly associated with DM [64]. Inflammatory signaling in cardiomyocytes usually occurs as an early response to myocardial injury and is accompanied by an increased formation of mitochondrial and cytosolic ROS. Increased oxidative stress and inflammation cause activation of nuclear transcription factor-κB (NF-κB), which is known to induce collagen and fibronectin synthesis and production of inflammatory cytokines [65]. NF-κB could induce overexpression of a wide range of proinflammatory cytokines including interleukins (such as TNFα, interleukin-1β, interleukin-6, and interleukin-8), chemokines (i.e., monocyte chemotactic protein-1 (MCP-1)), adhesion molecules (i.e., selectins and adhesion molecules: intercellular adhesion molecule-1 (ICAM-1) and vascular cell adhesion molecule- 1 (VCAM-1)) and the migration of leukocytes into the myocardium [65,66,67,68]. These effects have been observed not only in cardiomyocytes but also in coronary endothelial and smooth muscle cells, as well as in fibroblasts [55]. Increased production of proinflammatory mediators leading to fibroblast proliferation and activation is responsible for increased myocardial fibrosis which is an important contributor to increased myocardial stiffness in DCM. 

## 7. Coronary Microvascular Dysfunction in Diabetes

DM directly causes coronary microvascular dysfunction, characterized by diminished vasodilatory response to different stimuli. It is caused by several mechanisms: endothelial dysfunction per se (deranged ratio between local vasodilators, particularly low NO content, and vasoconstrictors), dysfunction of coronary smooth muscle cells, sympathetic dysfunction (increased α-adrenergic responses that are proconstricting), and consequent microvascular remodeling (replacement of functional with less functional tissue) [69,70]. The mentioned processes are based on local inflammation induced by hyperglycemia, mitochondrial fragmentation and dysfunction, ROS production, as well as nitrotyrosine production; these processes all lead to endothelial cell dysfunction, characterized by expression of VCAMs and selectins, leading to migration of leukocytes into the subendothelial space. Subendothelial leukocytes are responsible for further ROS production as well as potentiation of inflammation [70]. These mechanisms are similar to those described in the previous chapter. Together they damage the endothelial cells further and cause diminished NO bioavailability. Consequently, the vasodilatory response of the coronary vessels is reduced [69,71]. The latter, together with increased profibrotic cytokine signaling, may contribute to the reduced number of coronary arteries and increase in myocardial fibrosis, seen in DCM. The vicious cycle is thus completed. Coronary microvascular dysfunction, through described mechanisms, leads to microvascular ischemia. This, in turn, results in impaired coronary flow reserve that leads to cardiomyocyte injury and fibrosis, responsible for diastolic dysfunction [71]. In the milieu described, the processes are perpetuating one another, leading to overt heart failure. 

## 8. Various Transporters and Substrate Metabolism in Cardiomyocytes in Diabetes

Optimal functioning of myocardium depends on production of adequate amount of adenosine 5′-triphosphate (ATP), produced predominantly by mitochondrial oxidative phosphorylation and, to a lesser extent, glycolysis [72]. The most important source of energy in healthy myocardium is oxidation of FFA, as it provides up to 70% of the resynthesized ATP, but other substrates (glucose, lactate, amino-acids, and ketone bodies) can also be used for ATP production [72,73]. DM is associated with wide spectrum of myocardial metabolic derangements affecting both fuel supply and utilization. 

Glucose is hydrophilic, thus unable to travel through the cardiomyocyte plasma membrane by passive diffusion. Consequently, it is transported via two distinctive types of glucose transporters, i.e., facilitative glucose transporters (GLUT) and SGLTs [74]. GLUTs belong to a major facilitator superfamily that comprises 14 members. They are encoded by the *SLC2A* gene, and their expression is tissue-specific. The most abundant, i.e., 70% of all GLUT transporters in the heart, is GLUT-4. It is located mainly in intracellular membrane compartments and is translocated to the surface when stimulated, i.e., by insulin, hypoxia, catecholamines, etc., when it can increase glucose influx into the cardiomyocytes by 10- to 20-fold [75]. Additionally, GLUT-1 is also present in large amounts, its concentration falling from the neonatal period to adulthood. It is responsible for basal glucose transport and its expression is additionally stimulated by chronic hypoxia or long term fasting [76]. SGLTs, encoded by *SCL5A* genes (altogether 12), are all Na^+^/substrate cotransporters (transporting sugars, inositols, lactate, choline, urea, proline, and ions). Six genes are expressed in the human heart. The most expressed is SGLT-1, which colocalizes with GLUT-1 in the sarcolemma. It regulates the uptake of glucose due to hormonal stimuli [77]. On the other hand, SGLT-2s have not been found in human cardiomyocytes [78]. 

Insulin has been shown to affect transmembrane transport of glucose by increasing transcription of GLUT-1 and GLUT-4 transporters, promoting translocation of glucose transporter proteins to the plasma membrane and increasing their activity [79]. Thus, in the absence of insulin activity, due to either insulin deficiency or insulin resistance, myocardial glucose utilization is reduced. Since glucose cannot be utilized, there is a switch in substrate metabolism, particularly increasing the ATP production by FFA. The latter also causes insulin resistance and decrease in GLUT-4 availability, forming a vicious cycle [80]. On the other hand, there is an increase in SGLT-1 expression in diabetic hearts. This is thought to be a compensatory mechanism, due to reduction in cardiac expression of GLUT-1 and GLUT-4. This compensation is particularly seen in type 2 DM [77]. 

The FFAs are transported into the cardiomyocytes by passive diffusion (only a minor proportion) or through three distinct long chain FFA transporters, i.e., CD36, plasma membrane associated fatty acid-binding protein (FABP) and fatty acid transport protein (FATP) [81]. CD36 and FABP, CD36 acting solo or being the facilitator for the FABP, are responsible for the majority of the FFA uptake into the cardiomyocytes. These transporters form the functional pool, as they are located on the sarcolemma and responsible for energy uptake. Additionally, there is a storage pool localized in the intracellular compartments that can be recruited by various stimuli, i.e., contractile activity and insulin. When recruited, there is a vesicle mediated process that allows for the transporters to become functional [81,82]. In DM, there is an increased amount of CD36 in the sarcolemma, which is due to permanent relocation of this transport protein and not due to its increased expression. According to some authors, this is the key event in development of DCM [81]. 

Myocardial metabolism of FFA is impaired in DM due to increased circulating levels and increased FFA uptake due to upregulation and increased translocation of both CD36/FABP and FATP to sarcolemma [83]. β-oxidation of FFA is also reported to be increased in DM resulting in increased amount of acetyl-CoA, which inhibits pyruvate dehydrogenase and further decreases utilization of glucose and lactate in diabetic myocardium [72,73]. Increased β-oxidation also facilitates the transport of FFAs into the mitochondria, which is one of the most important regulatory steps of FFA metabolism [73]. When mitochondrial oxidative capacity is exceeded, excessive FFAs enter nonoxidative pathways, leading to production of toxic intermediates such as ceramide. Increased FFA oxidation in the mitochondria is associated with increased production of ROS, causing lipid peroxidation and impaired mitochondrial energy metabolism [84]. 

DM also affects the utilization of other substrates for energy metabolism: it decreases lactate uptake due to impaired pyruvate oxidation and increases the uptake of ketone bodies (KB) [73,85]. KB, i.e., acetoacetate and 3-β-hydroxybutyrate, are energy-rich compounds which are synthetized from FFAs in the liver. Insulin deficiency and increased amounts of counter regulatory hormones in DM are associated with increased ketogenesis due to increased transport of FFAs into mitochondria and their enhanced β-oxidation [86]. Excessive amounts of acetyl-CoA that cannot be included in the tricarboxylic acid (TCA) cycle are oxidized to form KB in hepatocytes. Because acetyl-CoA is generated through both ketone and FFA oxidation, there is a natural competition between ketones and FFAs for contribution to the TCA cycle, which is unaffected by DM [87]. In pathological conditions, such as poorly controlled DM, high levels of circulating KB inhibit not only the utilization of glucose and lactate but also the utilization of FFAs—the main substrates for energetic metabolism. Diabetic myocardium derives almost all ATP from FFA metabolism. Comparing to glycolysis, FFA utilization is ~10% less efficient at generating ATP after adjustment for oxygen consumption [88]. Consequently, impaired energy metabolism could lead to both contractile dysfunction and diastolic dysfunction, as it is also energy-consuming process. 

DM is also associated with increased catabolism of amino acids thus limiting the availability of amino acids for protein synthesis. Furthermore, DM is also associated with decreased RNA concentrations and inhibited protein synthesis, thus affecting not only structural proteins but also the contractile ones [89]. 

## 9. Sodium Glucose Cotransporter-2-Mediated Effects in Cardiomyocytes in Diabetes

Ion dysregulation, leading to deranged cardiomyocyte contraction and relaxation, is characteristic of diabetic myocardium. Particularly, Ca^2+^ and Na^2+^ homeostasis are altered. This is due to decreased Na^2+^/K^+^ pump activity and Na^2+^/Ca^2+^ exchanger (NCX) activity, with concomitantly increased Na^2+^/H^+^ exchanger (HNE) activity, leading to cytosol overload with Na^2+^ [90,91,92,93,94]. It has been found that there is increased SGLT-1 expression in diabetic failing myocardium, while SGLT-2 transporters were not found to be expressed in healthy or diseased cardiomyocytes. Favorable outcomes in recent cardiovascular outcome trials with SGLT-2 inhibitors were mostly highly selective for the inhibition of SGLT-2 (empagliflozin and dapagliflozin), and thus cannot be attributed to their effects on either SGLT [78]. Current knowledge indicates that this SGLT-2 inhibitors’ mediated effect on improvement of failing myocardium might be due to their direct inhibition of the HNE. This leads to decrease in intracellular Na^2+^ load and increased uptake of Ca^2+^ to the mitochondria and its efflux into the extracellular space (probably through NCX activity). Consequently, there is decrease in Ca^2+^ levels and better Ca^2+^ handling during the heart cycle [78,95,96,97]. Some data also suggests that SGLT-2 inhibitors directly increase phosphorylation of myofilament regulatory proteins, independently of glucose or Ca^2+^ metabolism modulation, thus reducing myofilament stiffness [98]. Additionally, diastolic dysfunction improvement with SGLT-2 inhibitors was also associated with its antifibrotic action that is mediated through the decrease of serum and glucocorticoid-regulated kinase-1 (SGK1)/Enac profibrotic pathway, which is otherwise highly expressed and activated in diabetic hearts [99]. 

On the other hand, dysregulation of metabolic pathways also plays an important role in failing diabetic myocardium. Due to insulin resistance, metabolism is switched to increased lipolysis and consequent fatty acid and triglyceride accumulation in the cardiomyocytes, which together with the inhibition of glucose oxidation, leads to myocardial steatosis and cytotoxicity [78]. Additionally, reduced glucose oxidation leads to glucose accumulation and formation of AGEs that are responsible for increased oxidative stress and cross-linking of collagen fibers contributing to myocardial stiffness and diastolic dysfunction [100,101,102]. Furthermore, mitochondrial dysfunction is present, characterized by reduced oxidative phosphorylation and reduced production of ATP. In conjunction with fatty acids accumulation, they enhance ROS production, leading to direct cytotoxicity and inflammation [103,104,105]. SGLT-2 inhibitors have been shown to directly switch myocardial energy consumption to KB and FFAs utilization while not leading to fatty acid accumulation. KB are more energy efficient, lead to less oxygen consumption, generate less ROS, possess antioxidant activities, and maintain mitochondrial integrity, thus influencing almost all of the above-mentioned pathophysiologic mechanisms resulting in increased myocardial stiffness [78,106,107,108,109].

## 10. Extracellular Matrix under Normal Physiology and in Pathophysiological States in Diabetic Myocardium

Cardiac ECM is a complex network of collagen and elastin fibers, different cell types (cardiomyocytes, fibroblasts, macrophages, leukocytes, etc.) as well as macromolecules such as glycoproteins and glycosaminoglycans together with growth factors, cytokines, and extracellular proteases [110]. ECM is essential for maintaining myocardial structure by connecting myocytes, aligning contractile elements, force transmission and preventing overextending and disruption of myocytes [24]. Nowadays, it has been recognized that ECM also has an important role in mechanosensing and mechanotransduction as well as in regulation of cytoskeleton stress and its morphology, as will be discussed in detail in the next section.

DM has profound effects on the expression, organization, and modification of ECM components in many organs [111]. Increased collagen deposition—particularly collagens type I and III—is one of the first morphological alterations of diabetic myocardium [10,11,17,20]. Fibroblasts represent approximately two-thirds of the cells in the myocardium and play a key role in the ECM turnover as they are involved in both synthesis and degradation of ECM components. Other populations of matrix-embedded cells may also contribute to the fibrotic process by modulating fibroblast phenotype and function [17]. As stated above, DM is associated with cardiomyocyte switching to fibrogenic phenotype, which is characterized by increased synthesis and release of cytokines that induce fibroblast proliferation and activation as well as proinflammatory mediators that trigger fibrosis through activation of immune cells: angiotensin II, TGFβ, TNFα, and interleukin-1 [17]. Increased collagen synthesis is probably the most important pathophysiological mechanism of cardiac fibrosis in DCM but increased proliferation potential of myocardial fibroblasts has also been reported in DM patients [111]. Finally, ECM degradation is also altered in DM due to decreased production and impaired activity of matrix metalloproteinases [67]. Decreased degradation of collagen fibers contributes to collagen accumulation and ECM fibrosis. 

Experimental evidence suggests that several different mediators could also promote DM-associated myocardial fibrosis including neurohumoral factors, inflammatory cytokines, and growth factors, endothelin-1, adipokines, and ROS [17]. Angiotensin-converting enzyme (ACE) inhibition is reported to be associated with reduction in collagen deposition and perivascular fibrosis in different rat models of DCM [112]. This suggests that increased activity of renin-angiotensin-aldosterone system (RAAS) observed in diabetic myocardium could be involved in the pathogenesis of DCM [113].

Increased myocardial fibrosis is the hallmark of DCM but probably all ECM components are affected by DM. Increased levels of AGEs in DM are promoting collagen cross-linking which, in turn, prevents enzymatic degradation of collagen [23,24]. Increasing evidence suggests that AGEs could also mediate inflammation, ROS generation, and fibrosis [17]. Changes in ECM proteoglycans content, a decreased level of heparan sulfate, and increased levels of chondroitin and dermatan sulfate have also been reported in DM [114].

## 11. Interactions between Cells and Extracellular Matrix in Diabetic Myocardium

Cells are continuously exposed to multiple external mechanical and chemical stimuli that modulate their structure and function. As opposed to external chemical stimuli, which have long been recognized as powerful modulators of cellular structure and function, external mechanical stimuli have recently been considered. Mechanical stimuli could be translated into biochemical signals by a process named mechanotransduction. The processes of mechanosensing and mechanotransduction in cardiomyocytes are complex as cardiomyocytes respond to external mechanical forces and generate internal mechanical forces as well. Cardiomyocytes have been shown to respond to different mechanical stimuli, including static and dynamic and compressive and tensile stress, as well as shear stress and substrate stiffness [115]. The mechanism by which cells sense ECM stiffness remains poorly understood. Some authors proposed that in a stationary cell firmly attached to ECM components, internal and external forces are equilibrated. Changing the force balance in either direction results in cell contraction, extension or translocation [116]. It has been proposed that fibroblasts can use their adhesion forces as sensors for the mechanical properties of their environment [117]. Based on the available data, it seems that most important factors for mechanosensing and mechanotransduction are integrins, focal adhesions, actomyosin contractility, and mechanosensitive ion channels [115,118]. 

Cytoskeleton is a complex structure responsible for maintaining cellular shape, internal organization, and stability; because of its connections to ECM components it can transmit mechanical stimuli both outside-in and inside-out. Transmission of the cytoskeleton forces to the ECM is facilitated by complexes of transmembrane proteins named integrins and their associated intracellular components. These complexes are also fundamental for sensing external mechanical forces [115]. Inside the cell, integrins are associated with proteins that link them to the actin cytoskeleton as well as signaling proteins included in modulation of cardiomyocyte contractility [119]. Outside the cell, integrins are associated with ECM proteins such as collagen, laminin, and fibronectin [120]. After binding of cardiac fibroblasts to ECM, integrins cluster to form complexes called focal adhesions, which are considered a primary mechanosensing organelle [121]. It has been shown that the size of focal adhesions depends on mechanical stress: substrate stiffness on the one hand and cytoskeletal forces on the other [122]. Besides integrins, numerous proteins participate in connections between the cytoskeleton and components of ECM such as dystrophin, sarcoglycans, dystroglycans, syntrophin, sarcospan, and caveolin 3 [123]. Mechanical stimuli are also transmitted to the nucleus of the cell [115]. It has been reported that mechanotransduction could modulate gene expression in fibroblasts [124].

ECM is widely recognized as a regulator of cytoskeleton stress and its morphology, and it has been demonstrated that cells modify and maintain their cytoskeleton according to mechanic stimuli from ECM [115]. Furthermore, as written above, isolated cardiomyocytes from diabetic rats expressed increased number and/or activation state of adhesion molecules on the cell surface [36]. This could probably alter the process of mechanosensing, leading to inappropriate or excessive cellular response to particular ECM stiffness. Thus, increased myocardial stiffness results from a positive feedback loop in which increased ECM stiffness induces changes in cardiomyocyte cytoskeleton, thus contributing to increased intrinsic cell stiffness and changing their phenotype to more fibrogenic. As a result, ECM fibrosis and ECM stiffness continue to increase, further affecting the cardiomyocyte cytoskeleton.

Mechanosensitive ion channels, particularly calcium-related, are also recognized as potential pathways by which cardiomyocyte could sense and respond to external mechanical loads. Increased stretching of cells is reported to cause downregulation of SERCA2 expression, thus limiting Ca^2+^ reuptake from the cytosol into the endoplasmic reticulum [125]. Consequently, increased stretching leads to increased intracellular Ca^2+^ concentration, thus causing increased cellular stiffness due to impaired relaxation [46,48]. Stretch is also reported to induce an increase in ROS in a process dependent on the membrane-bound NADPH oxidase 2 [126].

## 12. Potential Targets to Prevent and Treat Diastolic Dysfunction in Diabetes

The cytoskeletal properties that produce cell stiffness and lead to increased ECM stiffness with pathological interactions between them are elements that build a pathologic vicious cycle leading to diastolic dysfunction and heart failure. In order to prevent and treat DCM effectively, this cycle should be stopped. There are several points where possible successful interventions can be applicable. Large body of evidence shows that nonpharmacological interventions as well as lifestyle changes (weight control, smoking cessation, and aerobic exercise) are associated with favorable structural and functional cardiac changes and lower risk of heart failure in patients with DM [127,128]. Among them, aerobic exercise seems to play the most important role in preventing HF. Randomized control trials show that aerobic exercise improves myocardial systolic function and prevents systolic HF whereas the results regarding its impact on diastolic dysfunction are contradictory [128,129]. Meta-analyses show that aerobic exercise could improve diastolic dysfunction if started early in the disease course. It has also been shown that higher intensity of aerobic exercise is associated with improved diastolic function in patients with DM [127].

In addition to healthy lifestyle promotion, professional public’s interest is focused on the search for pharmacological solutions to prevent and treat diastolic dysfunction. The number of medications that are effective in systolic heart failure treatment and improve quality of life in those patients continues to increase. On the contrary, there is still no single medication for treatment of diastolic heart failure. Investigators tested widely used medications, such as aldosterone receptor antagonists, angiotensin II receptor antagonists, angiotensin convertase inhibitors, beta-blockers, calcium channel antagonists, statins, or their combinations, that were shown to be effective in diastolic dysfunction treatment in animal models, without any significant benefit [130,131,132,133].

It is well known that good glycemic control is one of the most important actions needed to prevent cardiovascular complications in DM [134]. Studies on rat models showed that good glycemic control is also associated with lower prevalence of DCM due to its association with reduced cardiomyocyte hypertrophy and reduced collagen deposition [135]. Knowing that, the first targets to prevent and treat DCM have been sought among antidiabetic medications. So far, some representative agents of the two bigger groups, namely SGLT-2 inhibitors and glucagon like peptide-1 receptor agonists (GLP-1RA) have shown benefits in this regard. As discussed earlier, some SGLT-2 inhibitors seem to have a direct effect on reducing myocardial stiffness, act antifibroticaly, switch myocardial energy consumption to ketone bodies, and thus act favorably in improving diastolic dysfunction [78,98,99,106,107,108,109]. For two of these agents, namely empagliflozin and canagliflozin, it has been shown in large clinical trials that they reduce major adverse cardiovascular events (MACE) by 14% compared to placebo [136,137]. Additionally, empagliflozin reduced the number of cardiovascular events by 38% [136], while this number was 13% for canagliflozin [137]. 

Similar efficacy in reducing MACE was shown for GLP-1RAs: liraglutide and semaglutide, namely for 13% and 26%, respectively, compared with placebo [138,139]. Liraglutide also reduced cardiovascular mortality for 22% [138], while this effect was not observed for semaglutide [139]. The mechanisms of these effects are not clear yet, but it seems they can be attributed to the improvement of endothelial dysfunction, reduction of blood pressure, increase in cardiomyocyte viability and inhibition of atherosclerosis [140,141,142,143,144]. 

As our understanding of pathophysiologic mechanisms underlying HFpEF continues to increase, more interest is focused towards medications that could interfere with some pathophysiologic processes leading to DCM. Thus, the lack of NO in cardiomyocytes and coronary endothelium is attracting particular interest. Riociguat and vericiguat are direct stimulators of soluble guanylate cyclase, leading to increased synthesis of cyclic guanosine monophosphate (cGMP), which increases NO bioavailability, resulting in systemic and pulmonary vasodilatation. Furthermore, riociguat has protective cardiovascular effects beyond increasing NO availability, as it reduces cardiac fibrosis, decreases left ventricular mass and atrial natriuretic peptide levels in animal models of hypertension [145]. The number of studies on their effect on HF is limited, but it has been showed that riociguat improves systolic function in patients with heart failure with reduced ejection fraction (HFrEF) [146]. On the other hand, initial study on the effect of vericiguat in patients with HFpEF showed improved quality of life but was not associated with improvement in plasma levels of N-terminal prohormone of brain natriuretic peptide (NT-proBNP) or with ultrasonographic parameters of diastolic dysfunction [147]. 

Inflammation plays an important role in pathogenesis of DCM, making medications used for reduction of systemic inflammation very interesting possible targets in prevention and treatment of DCM. Studies on cardiac function in patients with rheumatoid arthritis treated with IL-1 receptor blocker anakinra showed improved myocardial contractility and relaxation even after a single dose of medication. Furthermore, anakinra administration was associated with improved coronary flow reserve and brachial artery flow mediated dilatation (FMD) even up to 3 h after administration [148]. Another study showed that IL-1β blockade prevents deterioration of myocardial systolic and diastolic function after myocardial infarction in animal models [149]. The first randomized controlled trial investigating an association between anakinra and HFpEF in patients with increased parameters of systemic inflammation has not been finished yet. Investigators are comparing effects of anakinra (for 12 weeks) with placebo on clinical parameters, cardiovascular events, and ultrasonographic parameters of diastolic function as well as FMD. This study included only 30 patients but it will be very interesting to see its results [150]. As DM is characterized by increased systemic inflammation, it could be expected that anti-inflammatory drugs could have more prominent effect in those patients than in nondiabetic population. 

Most recently, one of the newest classes of drugs in HFrEF, namely angiotensin receptor-neprilysin inhibitors (ARNi), have also been proposed as treatment of HFpEF. Natriuretic peptides (NP) act cardioprotectively, by inhibiting RAAS and reducing sympathetic drive with additional antiproliferative and antihypertrophic effects. Degradation of NPs is processed by two mechanisms: neprilysin—a degrading enzyme—and NP receptor-mediated clearance. Consequently, neprilysin inhibition results in increased number of NPs. On the other hand, when RAAS is inhibited by angiotensin receptor blockers, this action is enhanced with NPs, thereby providing the rationale for combined blockade [151,152]. In HFpEF, e.g., DCM, ARNi seem to have their role as they increase the amount of cGMP in the cardiomyocytes through indirect activation of NP receptors. This leads to enhanced phosphorylation of titin, particularly its N2B isoform, and a consequent decrease in cardiomyocyte resting stiffness [152]. The effectiveness of ARNi in DCM has been shown recently in STZ rats, which were treated with the combination of telmisartan and thiorphan, the latter being a neprilysin inhibitor. Positive protective effects were shown in this study, namely through reduced inflammation, antifibrotic action, and antiapoptotic action with addition of reversal of histone acetylation in rat hearts [153]. Further studies in this field are needed in humans with HFpEF (currently ongoing) and particularly DCM.

## 13. Conclusions 

HF is the major cause of hospitalization and important predictor of increased mortality in patients with DM. DCM is important cause of HF in diabetic patients but its pathophysiology has not been completely understood so far. This is probably the main reason for the lack of effective preventive and treatment options.

In this article we propose the potential pathophysiological mechanisms responsible for myocardial hypertrophy and increased myocardial stiffness leading to diastolic dysfunction in patients with DM. Our model is based on the knowledge of vascular stiffness pathophysiology, as we believe that similar fundamental processes lead to myocardial stiffness. Based on the available data, it can be assumed that altered mechanical properties of myocardium in DCM are not solely attributable to changes in the ECM but also to changes in the intrinsic mechanical properties of cardiomyocytes. As those two entities interact in maintaining myocardial structure and function, we propose that their interactions also change in patients with DM thus further promoting development of both cellular and ECM stiffness. DM itself is probably the most important factor affecting cellular and ECM stiffness, but several other mechanisms should also be considered. Among them, the most important factors are impairment of myocardial NO pathway, coronary microvascular dysfunction, increased inflammation, and oxidative stress, and myocardium-SGLT-2-mediated effects. 

Better understanding of all factors involved in pathophysiology of development and progression of DCM would probably lead to development of new treatment options. The recently completed studies with new antidiabetic drugs, such as SGLT-2 inhibitors, which surprisingly somehow revealed large benefits in terms of improving heart failure in DM patients, shed a new light on an old problem. Better understanding of complex pathophysiology of DCM suggests considering antidiabetic, anti-inflammatory, and NO-stimulating drugs as potential new treatment options for DCM.

## Abbreviations

ACE	Angiotensin converting enzyme
AGE	Advanced glycation end product
ARNi	Angiotensin receptor-neprilysin inhibitor
ATM	Atomic force microscopy
ATP	Adenosine 5′-triphosphate
cGMP	Cyclic guanosine monophosphate
CIRKO	Cardiomyocyte selective insulin receptor knockout mice
CMRI	Cardiac magnetic resonance imaging
DCM	Diabetic cardiomyopathy
DM	Diabetes mellitus
e/n/iNOS	Endothelial/neuronal/inducible NO synthase
ECM	Extracellular matrix
FABP	Fatty acid-binding protein
FATP	Fatty acid transport protein
FFA	Free fatty acid
FMD	Flow mediated dilation
GK	Goto-Kakizaki rats
GLP-1RA	Glucagon like peptide-1 receptor antagonist
GLUT	Facilitative glucose transporters
HFD	High fat diet
HFpEF	Heart failure with preserved ejection fraction
HFrEF	Heart failure with reduced ejection fraction
HNE	Na^+^/H^+^ exchanger
ICAM-1	Intercellular cell adhesion molecule-1
KB	Ketone body
MACE	Major adverse cardiovascular events
MCP-1	Monocyte chemoattractant protein-1
MHC-PPARα	Mice with cardiomyocyte specific overexpression of the transcription factor for peroxisome proliferator activated receptor α
NADPH	Nicotinamide adenine dinucleotide phosphate
NCX	Na^+^/Ca^2+^ exchanger
NF-κB	Nuclear transcription factor-κB
NO	Nitric oxide
NOD	Non-obese diabetic mice
NP	Natriuretic peptide
NT-proBNP	N-terminal prohormone of brain natriuretic peptide
PET	Positron emission tomography
RAAS	Renin-angiotensin-aldosterone system
ROS	Reactive oxygen species
RT	Resting tension
SERCA2	Sarco/endoplasmic reticulum Ca^2+^-ATPase 2
SGK1	Serum and glucocorticoid-regulated kinase-1
SGLT	Sodium glucose cotransporter
SR	Sarcoplasmic reticulum
STZ	Streptozotocin
TCA	Tricarboxylic acid
TDI	Tissue doppler imaging
TGFβ	Transforming growth factor β
TNFα	Tumor necrosis factor α
VCAM-1	Vascular cell adhesion molecule-1
ZDF	Zucker diabetic fatty rats
